# Extraction of Dibenzyl Disulfide from Transformer Oils by Acidic Ionic Liquid

**DOI:** 10.3390/molecules29102395

**Published:** 2024-05-19

**Authors:** Lili Zhang, Pei Peng, Qian Pan, Fang Wan, Huaxin Zhang

**Affiliations:** Hubei Key Laboratory of Drug Synthesis and Optimization, Jingchu University of Technology, Jingmen 448000, China; zhangliligf@126.com (L.Z.);

**Keywords:** desulfurization, imidazolium-based ionic liquids, dibenzyl disulfide, extraction

## Abstract

In recent years, dibenzyl disulfide (DBDS) in transformer oils has caused many transformer failures around the world, and its removal has attracted more attention. In this work, nine imidazolium-based ionic liquids (ILs) were applied as effective, green desulfurization extractants for DBDS-containing transformer oil for the first time. The results show that the desulfurization ability of the ILs for DBDS followed the order of [BMIM]FeCl_4_ > [BMIM]N(CN)_2_ > [BMIM]SCN > [BMIM](C_4_H_9_O)_2_PO_2_ > [BMIM]MeSO_4_ > [BMIM]NTf_2_ > [BMIM]OTf > [BMIM]PF_6_ > [BMIM]BF_4_. Especially, [BMIM]FeCl_4_ ionic liquid had excellent removal efficiency for DBDS, with its S partition coefficient *K_N_* (S) being up to 2642, which was much higher than the other eight imidazolium-based ILs. Moreover, the extractive performance of [BMIM]FeCl_4_ increased with an increasing molar ratio of FeCl_3_ to [BMIM]Cl, which was attributed to its Lewis acidity and fluidity. [BMIM]FeCl_4_ ionic liquid could also avail in the desulfurization of diphenyl sulfide (DPS) from model oils. The experimental results demonstrate that π−π action, π-complexation, and Lewis acid−base interaction played important roles in the desulfurization process. Finally, the ([BMIM]FeCl_4_) ionic liquid could be recycled five times without a significant decrease in extractive ability.

## 1. Introduction

Transformer oils are essential for power transformers, which bear the function of insulation and heat dissipation [1]. Recently, the shunt reactors and power fault failures caused by corrosive sulfur have attracted increasing public attention [2,3]. The copper in the power transformer is easily corroded by corrosive sulfur, resulting in a reduction in the dielectric loss factor (tan δ) and a degeneration of metal and insulating paper [4,5]. When dibenzyl disulfide (DBDS) concentration is higher than 20 ppm, it becomes the main corrosive sulfur compound in the transformer oils. Specifications about DBDS content in transformer oils are becoming increasingly stringent worldwide. The content of DBDS in transformer oil was required to be less than 5 ppm by the new transformer oil standard IEC 60296 in 2012 [6]. Therefore, the removal of dibenzyl disulfide (DBDS) in transformer oils has become an urgent subject [7,8]. Various technologies have been employed to deal with DBDS, including adsorption desulfurization, oxidation desulfurization, extraction desulfurization, and so on [3,9,10]. Adsorption desulfurization and extraction desulfurization are economic and direct methods. Take the adsorption method, for example: silica gel and natural clays could be applied to remove DBDS from transformer oils, but their adsorption capacities for DBDS are relatively low, which limits their applications [11,12]. In comparison, extraction desulfurization has higher efficiency and has been widely used by many researchers. Some conventional extractants, such as polyalkyleneglycol, *N*, *N*-dimethylformamide, *N*-methyl pyrrolidone, and *N*-methylimidazole, have been employed to extract dibenzyl disulfide [13]. However, these solvents show a certain solubility in oil, resulting in loss of extractant and cross-contamination [14,15]. Therefore, it is of great importance to explore extractants that are environmentally friendly, highly efficient, and recyclable.

Ionic liquids (ILs) have been widely used as green solvents instead of organic solvents due to their nonvolatility, immiscibility, thermal stability, and customizability. Considering the above advantages, much attention has been paid to the application of ILs in extraction processes [16,17,18]. Imidazolium, pyrrolidinium, and pyridinium are typical cations for ILs, which are widely employed to construct ILs for extraction use. For example, imidazolium-based and pyrrolidinium-based ILs with different anions have recently been reported to extract thiophene and benzothiophene. It was found that the imidazolium-based ILs possessed the best extraction selectivity for thiophene and benzothiophene [19]. Imidazolium-based cationic ionic liquids (ILs) possessed good stability and fluidity, which is one of the essential requirements for high extraction efficiency. Favorable viscosity is an important factor in industrial application [20]. Based on the theory of hard and soft acids and bases, Gao employed inorganic Lewis’s acids to remove 3-methylthiophene and obtained excellent results [21]. Li et al. [16] proved that metal-based ILs have stronger interaction with the sulfur compound with theoretical and experimental evidence. They found that the desulfurization ability of metal-based ILs for dibenzothiophene (DBT) followed the order FeCl_3_ > AlCl_3_ > ZnCl_2_ > Cu(I)Cl. DBDS possessed stronger alkalinity than other corrosive sulfur compounds, which might be attributed to the increasing σ lone pair electron of S atoms. Lewis acidic ILs containing metal halide anions, such as FeCl_4_^−^, ZnCl_3_^−^, and AlCl_4_^−^, are active factors that affect the desulfurization results of ILs [22,23,24,25,26].

Hereby, 1-methylimidazolium was selected as the cation to construct ionic liquids (ILs) with Lewis acidic 1-butyl-3-methylimidazolium chloride/FeCl_3_. Nine ILs were synthesized and applied in DBDS extraction from transformer oil, which were expected to effectively solve the insulation failures caused by corrosive sulfur in transformer oil. To decipher the extractive mechanism, the interaction modes were analyzed via theoretic calculation methods.

## 2. Results and Discussion

### 2.1. Desulfurization Performance of ILs

**Figure 1:**
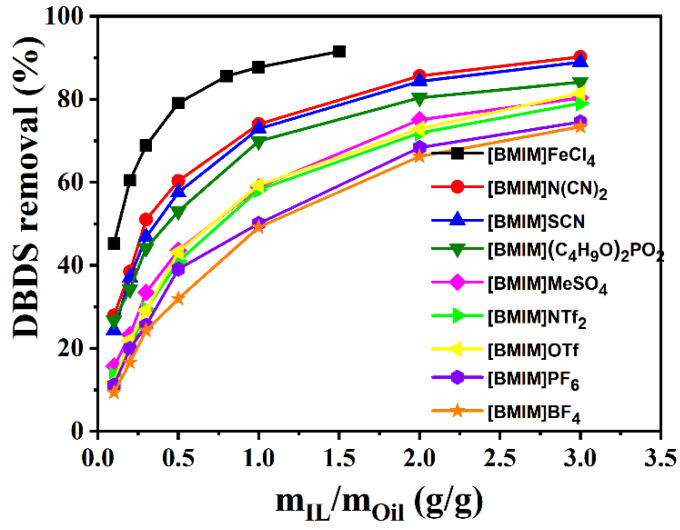
Comparison of desulfurization performance of imidazolium ILs. (T = 303 K; t = 15 min).

**Figure 2:**
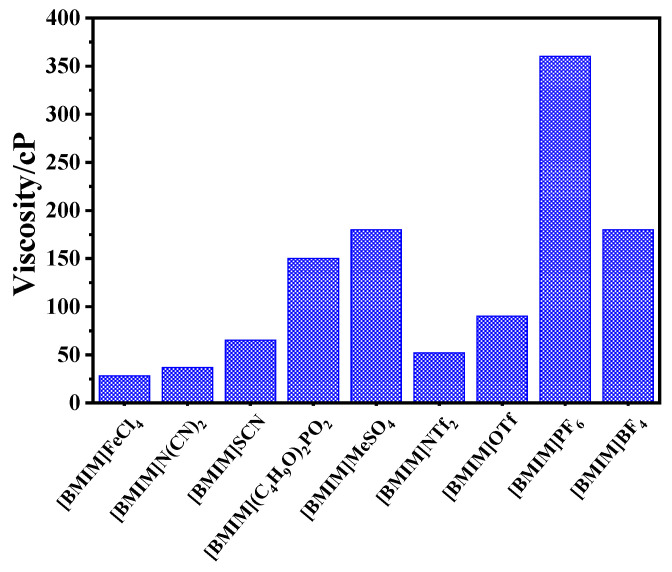
The viscosity of ILs.

In this work, DBDS extraction efficiency of nine imidazolium-based ionic liquids, including [BMIM]BF_4_, [BMIM]SCN, [BMIM]NTf_2_, [BMIM](C_4_H_9_O)_2_PO_2_, [BMIM]N(CN)_2_, [BMIM]MeSO_4_, [BMIM]OTf, [BMIM]PF_6_, and [BMIM]FeCl_4_, was determined. Figure 1 shows that desulfurization efficiency of the nine ILs followed the order [BMIM]FeCl_4_ > [BMIM]N(CN)_2_ > [BMIM]SCN > [BMIM](C_4_H_9_O)_2_PO_2_ > [BMIM]MeSO_4_ > [BMIM]NTf_2_ > [BMIM]OTf > [BMIM]PF_6_ > [BMIM]BF_4_. The order of ionic liquids viscosity in Figure 2 is consistent with the above experimental results, except for [BMIM]NTf_2_, [BMIM]OTf, and [BMIM]BF_4_. The lower the viscosity of the ionic liquid was, the more fully it could contact transformer oils and the higher the extraction efficiency it could achieve. This result indicates that the viscosity of ionic liquid played an important role in the extraction process. However, [BMIM]FeCl_4_ and [BMIM]N(CN)_2_ with similar viscosity showed large extraction differences, which might be caused by the differences in acidity.

**Figure 3:**
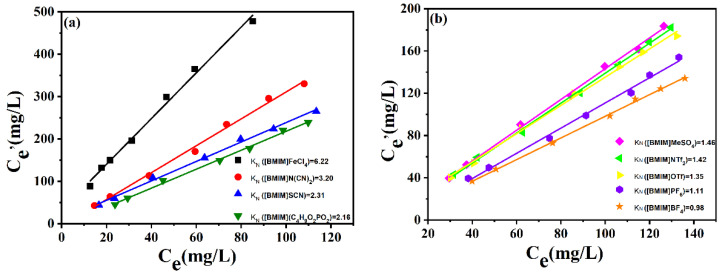
DBDS concentration in transformer oils and ILs. (**a**) [BMIM]FeCl_4_, [BMIM]N(CN)_2_, [BMIM]SCN, [BMIM](C_4_H_9_O)_2_PO_2_. (**b**) [BMIM]MeSO_4_, [BMIM]NTf_2_, [BMIM]OTf, [BMIM]PF_6_, [BMIM]BF_4_. (T = 303 K; t = 15 min).

*K_N_* referred to the concentration ratio of DBDS in extractant and in oil. For one IL, the *K_N_* value was not correlated with DBDS content in transformer oil when the DBDS content was low enough. Based on this, the *K_N_* value for each IL was measured using extraction oils with different DBDS content. As presented in Figure 3, the *K_N_* values displayed the same order as the DBDS removal efficiency list in Figure 1. [BMIM]FeCl_4_ showed the most excellent desulfurization performance; its *K_N_* value reached as high as 6.2. The *K_N_* values of [BMIM]OTf, [BMIM]PF_6_, and [BMIM]BF_4_ were only 1.35, 1.11, and 0.984 at 303 K, respectively. We could be certain that the difference in desulfurization efficiency of these ionic liquids containing imidazolium cation had a great relationship with the anions. Obviously, FeCl_4_^−^ was more propitious to DBDS extraction under experimental conditions. Moreover, the mass ratio of ionic liquids to oils also influenced DBDS removal rate. High ionic liquid/oil ratio could lead to a higher DBDS removal rate. As for [BMIM]FeCl_4_, the removal ratio of DBDS could amount to 90% when the mass ratio of [BMIM]FeCl_4_ to oil stood at 1.5. If deep desulfurization was needed, one could increase either the ratio of ILs to oils or the extraction times.

### 2.2. Effect of Molar Ratios of FeCl_3_/[BMIM]Cl on DBDS Extraction

**Figure 4:**
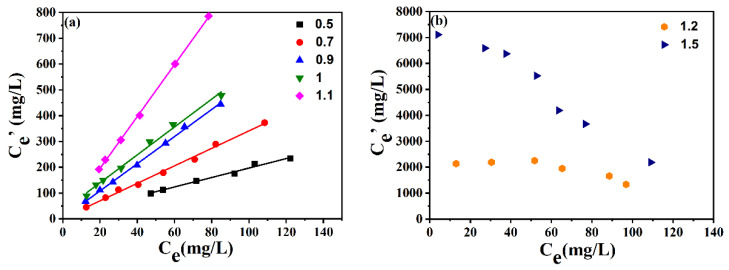
Effect of molar ratios of FeCl_3_/[BMIM]Cl on extraction efficiency. (**a**) Molar ratio at 0.5~1.1; (**b**) Molar ratio at 1.2 and 1.5

As stated above, [BMIM]FeCl_4_ presented the best desulfurization efficiency among the ILs. Thereby, [BMIM]FeCl_4_ was selected as the extractant to investigate the effect of ILs’ compositions on desulfurization performance. [BMIM]FeCl_4_ with different FeCl_3_/[BMIM]Cl ratios (0.5:1, 0.7:1, 0.9:1, 1:1,1.1:1, 1.2:1, 1.5:1) was discussed. As depicted in Figure 4, the desulfurization efficiency of ILs increased when the molar ratio of FeCl_3_ to [BMIM]Cl increased from 0.5:1 to 1.5:1. The *K_N_* value reached as high as 2642 when the FeCl_3_/[BMIM]Cl molar ratio increased to 1.5:1. The high *K_N_* value was due to the increased Lewis acidity of the IL at higher molar ratio of FeCl_3_/[BMIM]Cl.

### 2.3. Extraction Properties of ILs towards Other Sulfur Species

**Figure 5:**
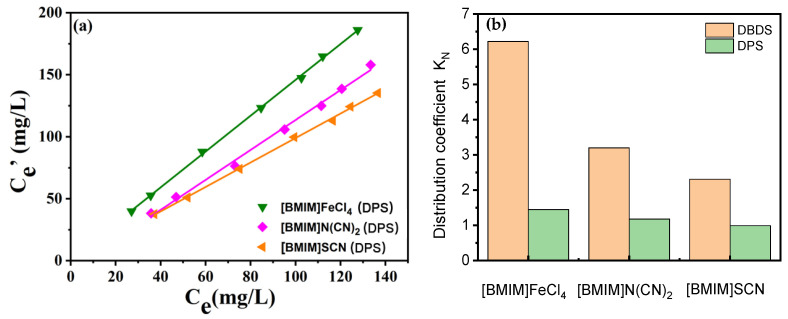
Effect of sulfur species on the extraction properties of [BMIM]FeCl_4_, [BMIM]N(CN)_2_ and [BMIM]SCN. (**a**) Extraction efficiency for DPS. (**b**) *K_N_* comparison of DBDS and DPS

To inspect the extraction performance of ILs towards other kind of sulfur species, diphenyl sulfide (DPS) was used, which was another common corrosive sulfur in transformer oils. The extraction experiments were carried out at room temperature, and the mass ratio of ILs and oils was 1:1. The results are presented in Figure 5. It was found that [BMIM]FeCl_4_ also showed the best removal efficiency for DPS. The other two ILs, [BMIM]N(CN)_2_ and [BMIM]SCN, exhibited lower extraction efficiency for DPS than [BMIM]FeCl_4_, indicating that FeCl_4_^−^ played an important role in extraction. It is worth noting that the extraction efficiency of [BMIM]FeCl_4_ towards DPS was much lower than that towards DBDS. The sulfur partition coefficient of DBDS in [BMIM]FeCl_4_ was 6 times as high as that of DPS, suggesting that sulfur species also affect the interaction. It was analyzed that the electron density of sulfur substances was the main reason for the difference in extraction efficiency. The higher π-electron density of DBDS contributed to the further enhancement of interaction between sulfur substances and extractant.

### 2.4. Determination of the Equilibrium Time

**Figure 6:**
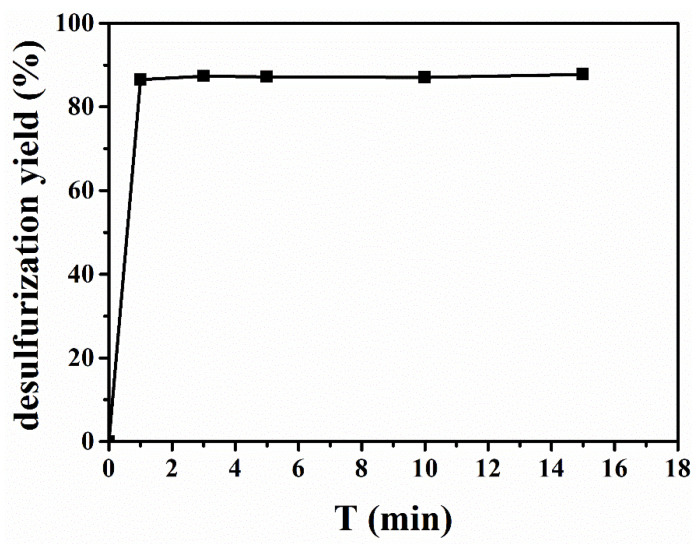
Determination of the equilibrium time for [BMIM]FeCl_4._

Figure 6 recorded the desulfurization efficiency of [BMIM]FeCl_4_ at 303 K at different times. The desulfurization efficiency was measured at 3, 5, 10, and 15 min at 303 K with the mass ratio of [BMIM]FeCl_4_ to oil at 1:1. As shown in Figure 6, the extraction process of [BMIM]FeCl_4_ reached equilibrium within 1 min, which was ascribed to low viscosity and strong acidity of [BMIM]FeCl_4_. On one hand, low viscosity of [BMIM]FeCl_4_ was beneficial to dispersion mass transfer. On the other hand, its strong acidity enhanced its chemical interaction with DBDS. Therefore, [BMIM]FeCl_4_ showed an excellent extractive rate, which is an important factor in the industrial extraction process.

### 2.5. Effect of Extraction Temperature on Desulfurization Performance of ILs

**Figure 7:**
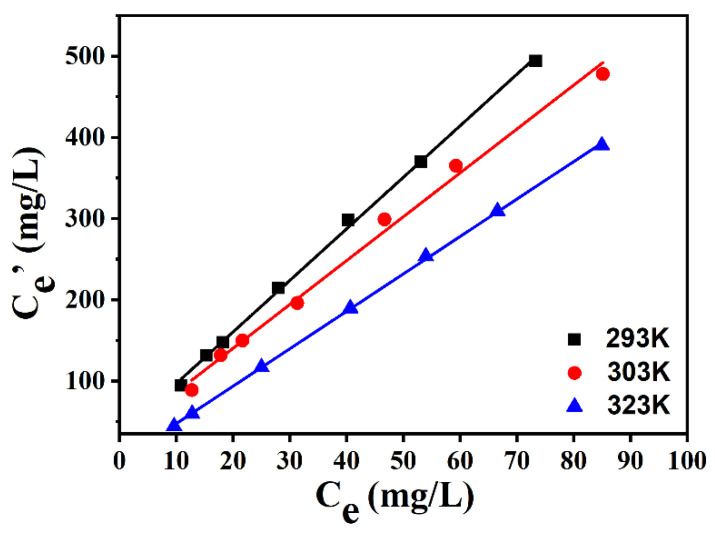
Desulfurization efficiency of [BMIM]FeCl_4_ at different temperatures.

Figure 7 displays the influence of the extraction temperature on the desulfurization efficiency of [BMIM]FeCl_4_ (1:1). The desulfurization performance of [BMIM]FeCl_4_ was obviously influenced by temperature. The *K_N_* of DBDS decreased when the temperature rose, which implied the extraction process was exothermic. Thereby, the desulfurization process of [BMIM] FeCl_4_ could be directly performed at room temperature, which would reduce energy consumption and the separation cost.

### 2.6. Comparison with Other Extractants

**Figure 8:**
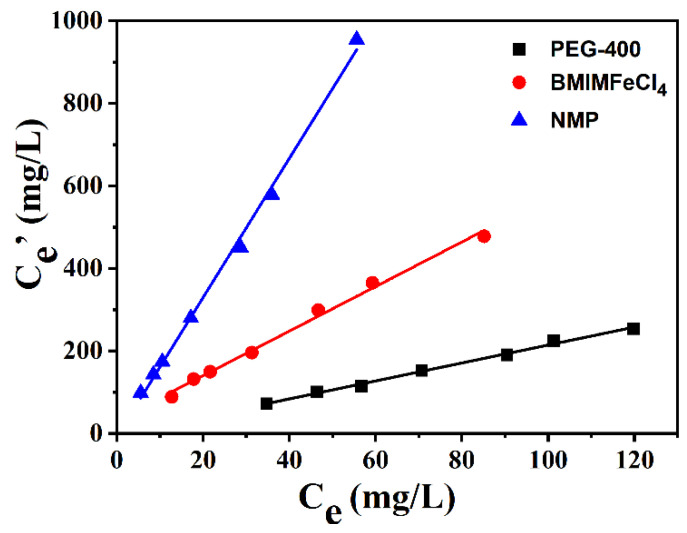
Desulfurization of ILs and other extractants for DBDS.

As stated above, [BMIM]FeCl_4_ presented high extraction efficiency for the DBDS. The *K_N_* of [BMIM]FeCl_4_ (1:1) could reach 6.2, which was superior to the commonly used extraction in industry. The comparison between [BMIM]FeCl_4_ and other extractants is shown in Figure 8. The *K_N_* for DBDS exhibited an order of *N*-methyl-pyrrolidone (NMP) > [BMIM]FeCl_4_ > PEG-400 at 303 K. The differences in desulfurization performance were ascribed to different polarity and surface tension properties [27]. The polarity and surface tension of NMP were much higher than that of [BMIM]FeCl_4_ and PEG-400, resulting in excellent desulfurization efficiency. But NMP has obvious drawbacks in industrial applications because of its large solubility in oil [28].

### 2.7. Accumulative Extraction of [BMIM]FeCl_4_

**Figure 9:**
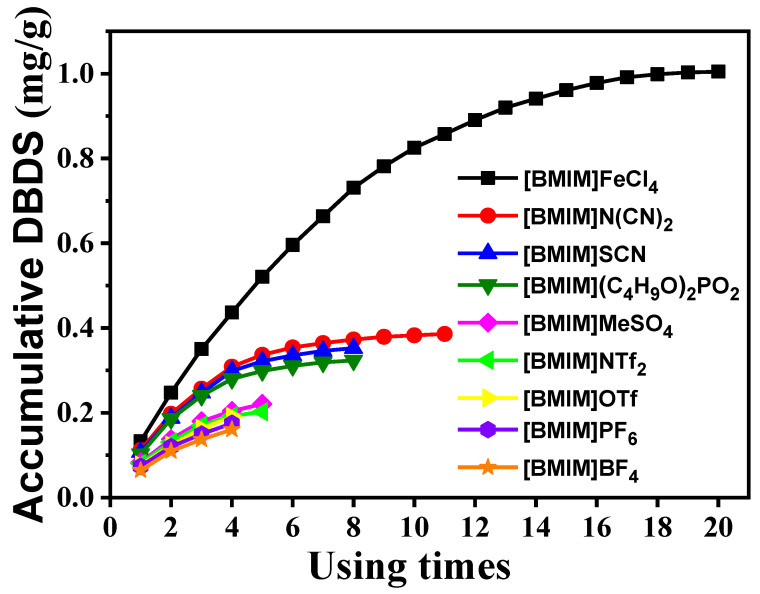
Accumulative extraction curve for DBDS by [BMIM]FeCl_4_ at 303 K.

The accumulative extraction experiment was carried out to evaluate desulfurization performance of [BMIM]FeCl_4_, as shown in Figure 9. It can be seen that the accumulative DBDS content of [BMIM]FeCl_4_ was much higher than that of other ILs. Moreover, DBDS content in [BMIM]FeCl_4_ kept rising during 20 accumulative extractions, which indicated that [BMIM]FeCl_4_ had a larger capacity for DBDS.

### 2.8. The Possible Extraction Mechanism

**Figure 10:**
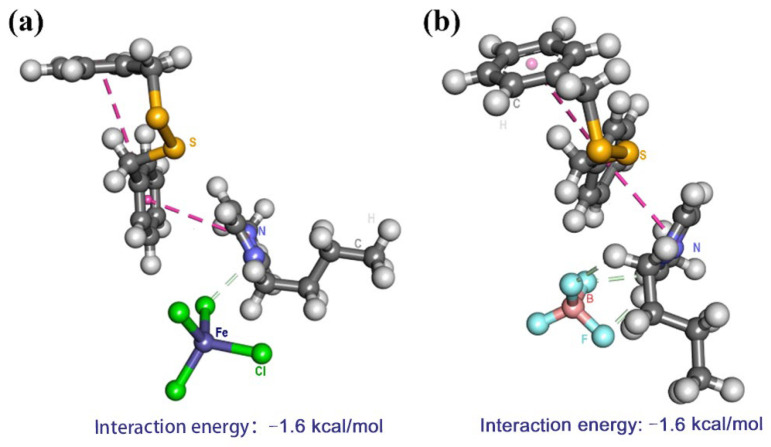
(**a**) Interaction energy between DBDS and [BMIM]FeCl_4_, (**b**) interaction energy between DBDS and [BMIM]BF_4_.

Based on extraction experiment results and characterization of imidazolium-based ionic liquids, π−π action and Lewis’s acid–base action were presumed to account for excellent desulfurization efficiency. For the imidazole cation, its π electron cloud could form π–π interactions with benzene ring on DBDS. Furthermore, the methyl and butyl substituents on the imidazole cation were electron-donating groups, which increased the polarizability and aromatic π-electron density of the imidazole cation. Therefore, the methyl and butyl substituents on the imidazolium ring could strengthen the interaction between ILs with DBDS through π–π stacking. The difference in desulfurization efficiency of these ILs was ascribed to anions. Compared to metal-free ILs, [BMIM]FeCl_4_ exhibited much higher desulfurization ability, which might be attributed to π-complexation and Lewis’s acid–base action interaction between [BMIM]FeCl_4_ and DBDS. Firstly, the anti-bonding π orbitals on DBDS would interact with the vacant *s* or *d* orbitals of Fe^3+^ (electron configuration: 1*s*^2^ 2*s*^2^ 2*p*^6^ 3*s*^2^ 3*p*^6^ 3*d*^5^ 4*s*^0^) via π-complexation interaction. The experimental results show that the extractive efficiency of ILs increased with the increasing of molar ratios of FeCl_3_/[BMIM]Cl. The higher FeCl_3_ content of ionic liquid gave stronger Lewis’s acidity and stronger acid–base action. Meanwhile, the interaction intensity between ILs ([BMIM]FeCl_4_, [BMIM]BF_4_) and DBDS was represented by interaction energy (ΔE, kJ·mol^−1^), as shown in Figure 10. The interaction energy (ΔE) was calculated as –1.6 kcal mol^−1^ between [BMIM]FeCl_4_ and DBDS, which was 0.5 kcal mol^−1^ lower than that between [BMIM]BF_4_ and DBDS. This was consistent with the experimental results. However, the interaction energy between [BMIM]FeCl_4_ and DBDS was higher than that between ethanethiol and [BMIM]^+^ (–10.92 kcal mol^−1^) [29], indicating that the attraction force of [BMIM]^+^ to DBDS was lower than that to ethanethiol. This allowed an easier back-extraction operation in the regeneration step.

### 2.9. Regeneration of [BMIM]FeCl_4_

**Figure 11:**
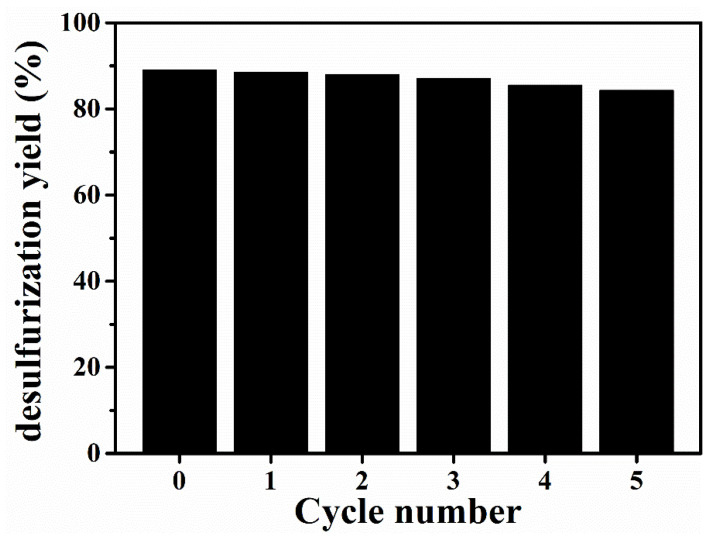
Regeneration performance of [BMIM]FeCl_4_ (*T* = 303 K; *m*_IL_/*m*_oiL_ = 1/1).

Regeneration and low-cost recycling were very important factors during the industrialization of extractants. To achieve the regeneration of [BMIM]FeCl_4_, organic solvent cyclohexane was used to separate DBDS from the [BMIM]FeCl_4_. The desulfurization efficiency of recycled [BMIM]FeCl_4_ is displayed in Figure 11. The desulfurization efficiency of [BMIM]FeCl_4_ remained at 85.39% after five cycles. Thereby, [BMIM]FeCl_4_ presented with good regeneration performance.

## 3. Material and Method

### 3.1. Materials

*N*-methylimidazole, iron (III) chloride anhydrous, 1-chlorobutane, Ethyl acetate, Dibenzyl disulfide (98%), n-hexadecane (98%), *N*-methyl-pyrrolidone (NMP), PEG-400, and cyclohexane were purchased from Macklin (Shanghai, China). 1-butyl-3-methylimidazolium dicyanamide [BMIM]N(CN)_2_, 1-butyl-3-methylimidazolium thiocyanate [BMIM]SCN, 1-butyl-3-methylimidazolium dibutyl phosphate [BMIM](C_4_H_9_O)_2_PO_2_, 1-butyl-3-methylimidazolium methyl sulfate [BMIM]MeSO_4_, 1-butyl-3-methylimidazolium bis((trifluoromethyl)sulfonyl)imide [BMIM]NTf_2_, 1-butyl-3-methylimidazolium trifluoromethanesulfonate [BMIM]OTf, 1-butyl-3-methylimidazolium hexafluorophosphate [BMIM]PF_6_, and 1-butyl-3-methylimidazolium tetrafluoroborate [BMIM]BF_4_ were provided by Shanghai Cheng Jie Chemical Co., Ltd. (Shanghai, China). All reagents were of analytical grade.

### 3.2. Preparation of [BMIM]Cl·xFeCl_3_

[BMIM]Cl·*x*FeCl_3_ ionic liquids were synthesized according to reference [30]. Certain amounts of anhydrous FeCl_3_ (*x* mol, *x* = 0.5, 0.7, 0.9, 1.0, 1.1, 1.2, 1.5) were mixed with [BMIM]Cl (1 mol) and stirred for 24 h at room temperature. Finally, a reddish-brown liquid, [BMIM]Cl·*x*FeCl_3_, was obtained.

### 3.3. Extractive Desulfurization Experiment

The model oil was prepared by dissolving DBDS in n-hexadecane. The content of DBDS was set at 150 ppm. Extraction processes were performed in a series of 10 mL tubes. Ionic liquids with varying molar compositions were added into model oil containing 150 ppm of DBDS. Then, the mixtures were stirred for 30 min at 303 K. After extraction, the mixture was separated after standing for 30 min. The oil sample was analyzed using a Wufeng EX1600 high-performance liquid chromatography (HPLC) system equipped with an LC-UV100^plus^ detector at 215 nm and a C18-100-5 4E column to determine the concentration of DBDS in the oil sample. The mobile phase in HPLC was 90% methanol aqueous with a rate of 1 mL/min.

### 3.4. Separation Parameter Determination

According to the DBDS content in the extractant and in the oil, extraction efficiency (*S-removal*) and distribution coefficient (*K_N_*) could be calculated using the following equations:

(1) $$S\text{-}removal=\frac{C_{0}-C_{e}}{C_{0}}\times 100$$

(2) $$K_{N}=\frac{{C_{e}}^{\prime }}{C_{e}}=\frac{\left(m_{0}C_{0}-m_{e}C_{e}\right)/{m_{e}}^{\prime }}{C_{e}}$$

where *C*_0_ is the DBDS content in the original oil, and *C*_e_ is the DBDS content in the oil phase after extraction. *C_e_′* and *C_e_* are the DBDS content in the extractant phase and oil, respectively. *m*_0_, *m_e_*, and *m_e_′* are the mass of the original oil, the oil phase after extraction, and the extractant phase after extraction, respectively.

### 3.5. Theoretical Calculation

To comprehend the desulfidation mechanism theoretically, the interactions between [BMIM]FeCl_4_ and DBDS were analyzed using the Gaussian 09 program [30,31,32]. The Cartesian coordinates of optimized structures, energies of ion pairs of RTILs, the optimized structures, and the interaction energies were calculated at uωb97xd/6-311 + g(d,p) level (see Supplementary Materials).

### 3.6. Regeneration of [BMIM]FeCl_4_

To check the reusability of [BMIM]FeCl_4_, the cyclohexane was chosen to re-extract DBDS after extraction. Cyclohexane was added to the extractant phase and stirred for 30 min at 303 K. The regenerated IL was separated from cyclohexane via a separatory method using a separating funnel. Subsequently, the IL containing trace cyclohexane was evaporated under 70 °C under reduced pressure for 3 h. Then, the recovered IL was used for the next cycle in extraction desulfurization.

## 4. Conclusions

In this work, a series of imidazolium-based ionic liquids with difference anions were used in the extraction of dibenzyl disulfide (DBDS) from transformer oils. [BMIM]FeCl_4_ (1:1.5) showed the highest efficiency in DBDS extraction, whose S partition coefficient *K_N_* reached as high as 2642. This benefited from the special Lewis acidity and polarity of [BMIM]FeCl_4_. The extraction performance of [BMIM]FeCl_4_ for DBDS was superior to that for DPS. The partition coefficient of DBDS in [BMIM]FeCl_4_ and oil was 6 times higher than that of DPS, which was due to the difference in electron density on the sulfur atoms in DBDS and DPS. The dominant interactions were assumed to be π−π action and Lewis’s acid−base interaction between [BMIM]FeCl_4_ and DBDS. Bearing favorable recyclability and high extraction efficiency, [BMIM]FeCl_4_ may be used as a potential extractant for the extraction of DBDS from transformer oils if the costs are further controlled.

## Data Availability

The original contributions presented in the study are included in the article/supplementary material, further inquiries can be directed to the corresponding authors.

## Supplementary Materials

The following supporting information can be downloaded at: https://www.mdpi.com/article/10.3390/molecules29102395/s1, Calculation details including tables showing the Cartesian coordinates of optimized structures, calculated energies of ion pairs of RTILs, and figures showing the optimized structures.
