# Supplementary material for: Extraction of Dibenzyl Disulfide from Transformer Oils by Acidic Ionic Liquid
Source: Molecules. 2024 May 19;29(10):2395. doi: 10.3390/molecules29102395 (PMC11124192; doi:10.3390/molecules29102395)

Supporting Information

Index

S1. The Cartesian coordinates of optimized structures 2

S1.1 [BMIM]$FeCl_4$ 2

S1.2 DBDS 2

S1.3 [BMIM]$FeCl_4$-DBDS 3

S1.4 [BMIM]BF_4 5

S1.5 [BMIM]BF_4-DBDS 6

S2 Calculated energy Data 8

S2.1 [BMIM]$FeCl_4$ 8

S2.2 DBDS 8

S2.3 [BMIM]$FeCl_4$-DBDS 9

S2.4 [BMIM]BF_4 10

S2.5 [BMIM]BF_4-DBDS 10

S3. Figures of the optimized structures 12

S3.1 [BMIM]$FeCl_4$-DBDS 12

S3.2 [BMIM]BF_4-DBDS 14

S1. The Cartesian coordinates of optimized structures

S1.1 [BMIM]$FeCl_4$

(The atomic symbol followed by three Cartesian coordinates, in Å).

C	-1.36992	0.878003	0.330328
C	-1.89719	2.818938	-0.55102
C	-2.3932	1.844745	-1.3527
N	-2.05934	0.641021	-0.78201
H	-1.92875	3.890783	-0.64023
H	-2.94657	1.903905	-2.27352
N	-1.27465	2.194177	0.501576
C	-0.46944	2.861177	1.520609
H	0.547318	2.979333	1.144656
H	-0.91947	3.828614	1.738533
H	-0.44686	2.249491	2.418889
C	-2.33374	-0.68518	-1.35948
C	-2.92249	-1.65136	-0.34271
H	-1.39392	-1.06408	-1.76322
H	-3.0314	-0.51418	-2.18163
H	-3.03614	-2.61325	-0.85261
H	-2.1936	-1.83169	0.454526
H	-0.92454	0.135061	0.974369
Fe	2.047328	-0.39663	-0.01448
Cl	1.598528	0.168603	2.098879
Cl	0.71178	-2.12258	-0.45199
Cl	1.381891	1.30039	-1.30916
Cl	4.146251	-0.85865	-0.31616
C	-4.26276	-1.20497	0.236881
H	-4.15006	-0.23005	0.725983
H	-4.97891	-1.05736	-0.58044
C	-4.82475	-2.2098	1.238084
H	-4.1379	-2.35312	2.077055
H	-5.78242	-1.87386	1.641832
H	-4.98217	-3.185	0.768768

S1.2 DBDS

C	-4.3896	-0.44696	0.354028
C	-3.13339	-0.9179	0.714141
C	-1.9887	-0.1719	0.433101
C	-2.12421	1.051235	-0.22273
C	-3.37964	1.524583	-0.58275

C	-4.51545	0.776063	-0.29542
H	-5.27168	-1.03479	0.581935
H	-3.03649	-1.87342	1.220008
H	-1.23528	1.633079	-0.44423
H	-3.47207	2.480066	-1.0871
H	-5.49585	1.145064	-0.57499
C	-0.6275	-0.68284	0.808331
H	-0.64782	-1.22741	1.753037
H	0.092513	0.131594	0.87864
S	-0.06491	-1.85159	-0.50324
S	1.831862	-2.36352	0.144659
C	2.892366	-1.09771	-0.67095
H	3.899634	-1.35662	-0.33301
H	2.841995	-1.25558	-1.74857
C	2.540011	0.315698	-0.31262
C	2.859771	0.836523	0.942593
C	1.837389	1.113733	-1.21281
C	2.475139	2.122301	1.293974
H	3.397802	0.219008	1.65505
C	1.45298	2.404623	-0.8639
H	1.575292	0.716174	-2.1873
C	1.765169	2.90999	0.391455
H	2.726794	2.51219	2.273838
H	0.906179	3.013904	-1.5753
H	1.461757	3.913762	0.666534

S1.3 [BMIM]$FeCl_4$-DBDS

C	2.27327	-1.40372	-0.05954
C	0.992915	-1.70727	-0.51866
C	0.093927	-2.39227	0.290415
C	0.468744	-2.77697	1.572004
C	1.740465	-2.46555	2.047188
H	0.686499	-1.40506	-1.51483
H	-0.90031	-2.61405	-0.07692
H	-0.23033	-3.32892	2.191536
H	2.039865	-2.77108	3.044089

Fe	-4.20459	-1.32071	-0.65799
Cl	-5.28463	0.576797	-0.16799
Cl	-2.39411	-0.77546	-1.83583
Cl	-3.50435	-2.1852	1.273058
Cl	-5.47191	-2.70927	-1.74568
C	-2.23461	1.183828	1.271555
C	-0.30318	1.603598	0.317593
H	-3.29732	1.129692	1.450903
C	-0.06853	0.832329	1.408012
H	0.378291	1.987312	-0.42199
H	0.845048	0.406786	1.789603
N	-1.28831	0.58772	1.992276
N	-1.65887	1.815394	0.253231
C	-1.50817	-0.19497	3.201199
H	-1.07068	0.325145	4.054012
H	-2.57786	-0.3281	3.343942
H	-1.04766	-1.1728	3.072763
C	-2.36649	2.585074	-0.78073
C	-3.10038	3.783755	-0.19681
H	-3.05004	1.903431	-1.28633
H	-1.60368	2.897733	-1.49564
H	-3.62547	4.271079	-1.0247
H	-3.88267	3.430473	0.484494
C	-2.18976	4.786519	0.507466
H	-1.65909	4.289852	1.328368
H	-1.41877	5.128239	-0.19353
C	-2.95697	5.986894	1.053601
H	-2.28862	6.690738	1.554783
H	-3.46834	6.524603	0.250078
H	-3.71548	5.67169	1.776113
C	3.247337	-0.65875	-0.92696
H	4.266887	-1.01204	-0.77567
H	2.988042	-0.73764	-1.98245
S	3.182572	1.122991	-0.45409
S	4.58139	2.004541	-1.69448
C	6.128494	1.897045	-0.69873
H	6.881308	2.333774	-1.36076

H	6.020418	2.545347	0.171128
C	6.491988	0.498945	-0.29554
C	6.266412	0.065278	1.009113
C	7.003501	-0.40497	-1.22817
C	6.532278	-1.25084	1.37344
H	5.866997	0.759367	1.740936
C	7.268966	-1.7181	-0.86733
H	7.174885	-0.07944	-2.2494
C	7.027559	-2.14697	0.435108
H	6.351651	-1.57571	2.392275
H	7.661819	-2.4109	-1.60277
H	7.23001	-3.17413	0.715936
C	2.637281	-1.78211	1.235175
H	3.635634	-1.5508	1.593357

S1.4 [BMIM]BF_4

C	-0.07361	0.752325	0.312672
C	0.177294	2.839814	-0.3212
C	-0.70621	2.248314	-1.16235
N	-0.85087	0.943576	-0.74659
H	0.569993	3.841548	-0.30056
H	-1.23541	2.636392	-2.01527
N	0.554838	1.8863	0.596052
C	1.608937	2.013253	1.597687
H	2.535319	2.284217	1.093753
H	1.328795	2.765581	2.335244
H	1.751224	1.040235	2.063804
C	-1.59041	-0.11451	-1.45432
C	-2.29212	-1.07527	-0.50663
H	-0.86626	-0.65019	-2.0691
H	-2.31107	0.396741	-2.09664
H	-2.76695	-1.83771	-1.13258
H	-1.54085	-1.60951	0.083144
H	0.092125	-0.18076	0.828159

C	-3.33916	-0.42193	0.392953
H	-2.87125	0.356691	1.006383
H	-4.08579	0.085963	-0.22953
C	-4.02943	-1.43284	1.303915
H	-3.30503	-1.93201	1.953652
H	-4.77469	-0.94997	1.940105
H	-4.5374	-2.20525	0.719311
B	2.12246	-1.31116	-0.1152
F	2.495543	-0.01178	-0.54503
F	1.771497	-1.21309	1.275404
F	0.933307	-1.67721	-0.81243
F	3.126925	-2.22572	-0.30978

S1.5 [BMIM]BF_4-DBDS

C	1.060093	-1.28452	0.172639
C	-0.11751	-1.77429	-0.38811
C	-0.67716	-2.96055	0.07486
C	-0.08029	-3.65279	1.121084
C	1.087068	-3.15941	1.69934
H	-0.60806	-1.23973	-1.19507
H	-1.58295	-3.32236	-0.39797
H	-0.5097	-4.58408	1.476472
H	1.563839	-3.70017	2.50992
C	-2.97318	-0.18	0.782768
C	-1.33977	0.252316	2.187167
H	-3.67408	-0.10551	-0.03873
C	-1.88362	-0.93848	2.537258
H	-0.50824	0.797357	2.597184
H	-1.61442	-1.6365	3.310145
N	-2.90317	-1.18751	1.646662
N	-2.03505	0.708452	1.089914
C	-3.73806	-2.38028	1.588824
H	-4.55773	-2.30267	2.303981
H	-4.11657	-2.47813	0.572046

H	-3.11815	-3.24537	1.821205
C	-1.71637	1.906238	0.294187
C	-2.95929	2.635588	-0.19215
H	-1.11549	1.575181	-0.55349
H	-1.10198	2.540663	0.934625
H	-2.60587	3.475749	-0.79892
H	-3.51789	1.990406	-0.87677
C	-3.86204	3.153472	0.925515
H	-4.1958	2.318309	1.552356
H	-3.28724	3.818808	1.581314
C	-5.08312	3.888834	0.380964
H	-5.72422	4.248701	1.189267
H	-4.78439	4.752741	-0.22
H	-5.67921	3.231076	-0.25753
C	1.699542	-0.04051	-0.38272
H	2.699089	-0.25248	-0.76308
H	1.09645	0.383667	-1.18564
S	1.867668	1.219659	0.952631
S	2.85064	2.777012	0.014121
C	4.625813	2.361791	0.294237
H	5.154781	3.168236	-0.22088
H	4.830507	2.443055	1.36202
C	5.013018	1.014876	-0.23841
C	5.134001	-0.0714	0.62605
C	5.180835	0.809422	-1.60872
C	5.393116	-1.34483	0.130218
H	5.003809	0.078883	1.692592
C	5.445013	-0.45889	-2.10514
H	5.0792	1.647116	-2.29147
C	5.543012	-1.54234	-1.2363
H	5.472559	-2.18413	0.811993
H	5.56621	-0.60614	-3.17225
H	5.737092	-2.53529	-1.62511
C	1.6574	-1.98713	1.222834
H	2.580948	-1.6169	1.656333
B	-3.36547	-0.80923	-2.33051
F	-4.45808	-0.07583	-1.76972

F	-3.22663	-1.99888	-1.55689
F	-3.57093	-1.08858	-3.65691
F	-2.19233	-0.02454	-2.13476

S2 Calculated energy Data

S2.1 [BMIM]$FeCl_4$

Imaginary Freq	0	
Temperature	298.15	Kelvin
Pressure	1	atm
Frequencies scaled by	1	
Electronic Energy (EE)	-3528.1235	Hartree
Zero-point Energy Correction	0.23366	Hartree
Thermal Correction to Energy	0.253988	Hartree
Thermal Correction to Enthalpy	0.254932	Hartree
Thermal Correction to Free Energy	0.177442	Hartree
EE + Zero-point Energy	-3527.8898	Hartree
EE + Thermal Energy Correction	-3527.8695	Hartree
EE + Thermal Enthalpy Correction	-3527.8686	Hartree
EE + Thermal Free Energy Correction	-3527.946	Hartree
E (Thermal)	159.38	kcal/mol
Heat Capacity (Cv)	66.728	cal/mol-kelvin
Entropy (S)	163.091	cal/mol-kelvin

S2.2 DBDS

Imaginary Freq	0	
Temperature	298.15	Kelvin
Pressure	1	atm

Frequencies scaled by	1	
Electronic Energy (EE)	-1338.27	Hartree
Zero-point Energy Correction	0.24297	Hartree
Thermal Correction to Energy	0.257643	Hartree
Thermal Correction to Enthalpy	0.258587	Hartree
Thermal Correction to Free Energy	0.198447	Hartree
EE + Zero-point Energy	-1338.03	Hartree
EE + Thermal Energy Correction	-1338.02	Hartree
EE + Thermal Enthalpy Correction	-1338.02	Hartree
EE + Thermal Free Energy Correction	-1338.08	Hartree
E (Thermal)	161.673	kcal/mol
Heat Capacity (Cv)	55.711	cal/mol-kelvin
Entropy (S)	126.574	cal/mol-kelvin

S2.3 [BMIM]$FeCl_4$-DBDS

Imaginary Freq	0	
Temperature	298.15	Kelvin
Pressure	1	atm
Frequencies scaled by	1	
Electronic Energy (EE)	-4866.42	Hartree
Zero-point Energy Correction	0.47756	Hartree
Thermal Correction to Energy	0.514694	Hartree
Thermal Correction to Enthalpy	0.515638	Hartree
Thermal Correction to Free Energy	0.395927	Hartree
EE + Zero-point Energy	-4865.94	Hartree
EE + Thermal Energy Correction	-4865.91	Hartree
EE + Thermal Enthalpy Correction	-4865.9	Hartree
EE + Thermal Free Energy Correction	-4866.02	Hartree
E (Thermal)	322.975	kcal/mol
Heat Capacity (Cv)	128.141	cal/mol-kelvin

Entropy (S)	251.953	cal/mol-kelvin

S2.4 [BMIM]BF_4

Imaginary Freq	0	
Temperature	298.15	Kelvin
Pressure	1	atm
Frequencies scaled by	1	
Electronic Energy (EE)	-847.842	Hartree
Zero-point Energy Correction	0.243076	Hartree
Thermal Correction to Energy	0.260028	Hartree
Thermal Correction to Enthalpy	0.260972	Hartree
Thermal Correction to Free Energy	0.195263	Hartree
EE + Zero-point Energy	-847.599	Hartree
EE + Thermal Energy Correction	-847.582	Hartree
EE + Thermal Enthalpy Correction	-847.581	Hartree
EE + Thermal Free Energy Correction	-847.647	Hartree
E (Thermal)	163.17	kcal/mol
Heat Capacity (Cv)	59.533	cal/mol-kelvin
Entropy (S)	138.296	cal/mol-kelvin

S2.5 [BMIM]BF_4-DBDS

Imaginary Freq	0	
Temperature	298.15	Kelvin
Pressure	1	atm
Frequencies scaled by	1	
Electronic Energy (EE)	-2186.14	Hartree
Zero-point Energy Correction	0.487777	Hartree

Thermal Correction to Energy	0.521049	Hartree
Thermal Correction to Enthalpy	0.521993	Hartree
Thermal Correction to Free Energy	0.417207	Hartree
EE + Zero-point Energy	-2185.65	Hartree
EE + Thermal Energy Correction	-2185.62	Hartree
EE + Thermal Enthalpy Correction	-2185.62	Hartree
EE + Thermal Free Energy Correction	-2185.72	Hartree
E (Thermal)	326.963	kcal/mol
Heat Capacity (Cv)	120.681	cal/mol-kelvin
Entropy (S)	220.54	cal/mol-kelvin

S3. Figures of the optimized structures

S3.1 [BMIM]$FeCl_4$-DBDS

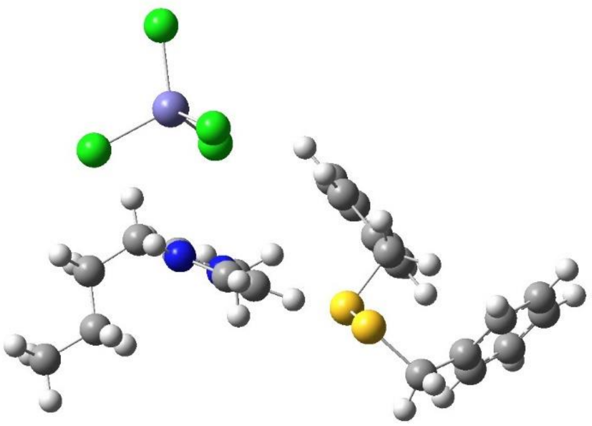

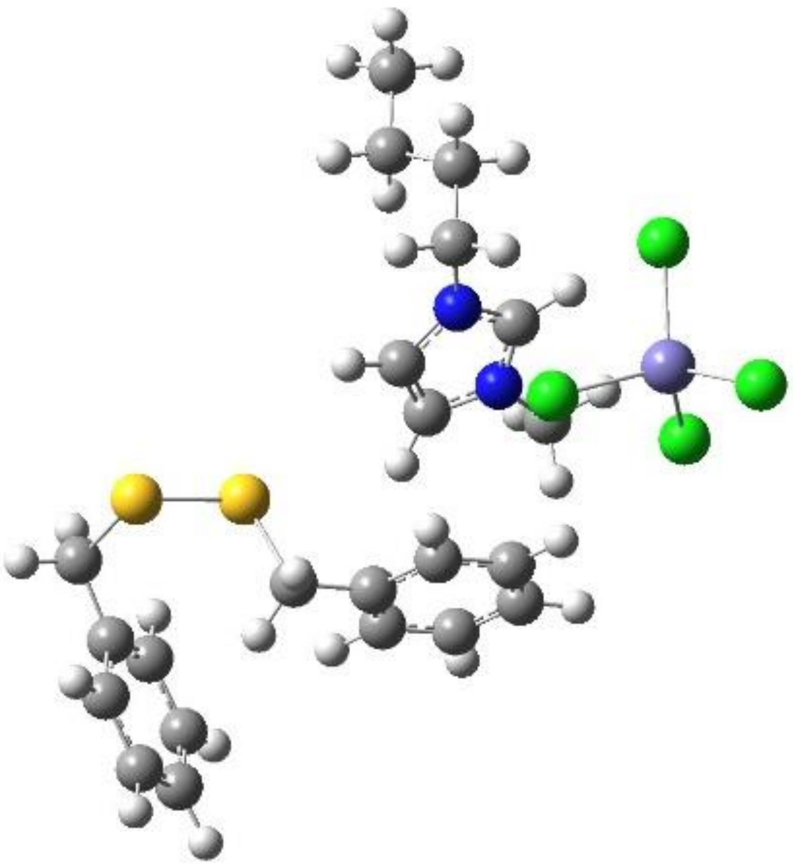

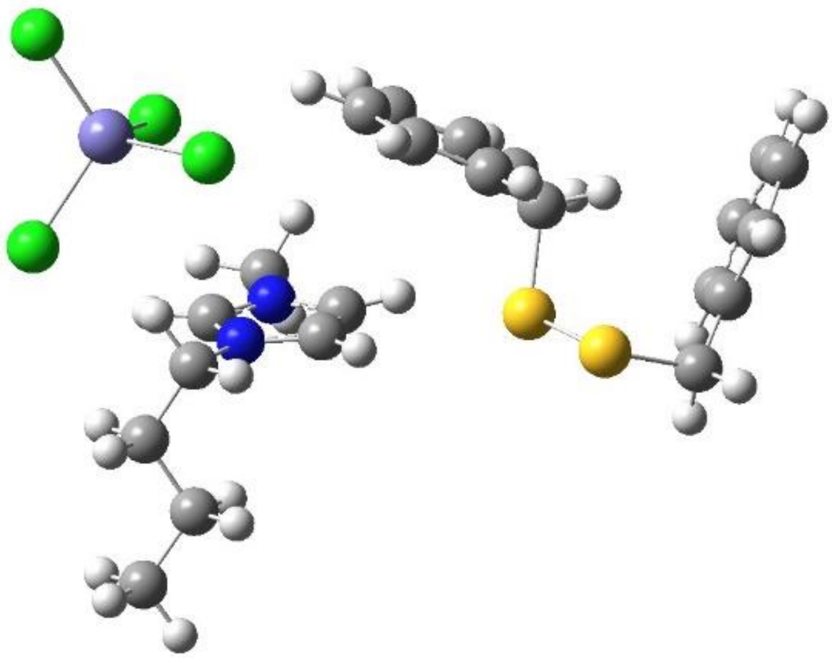

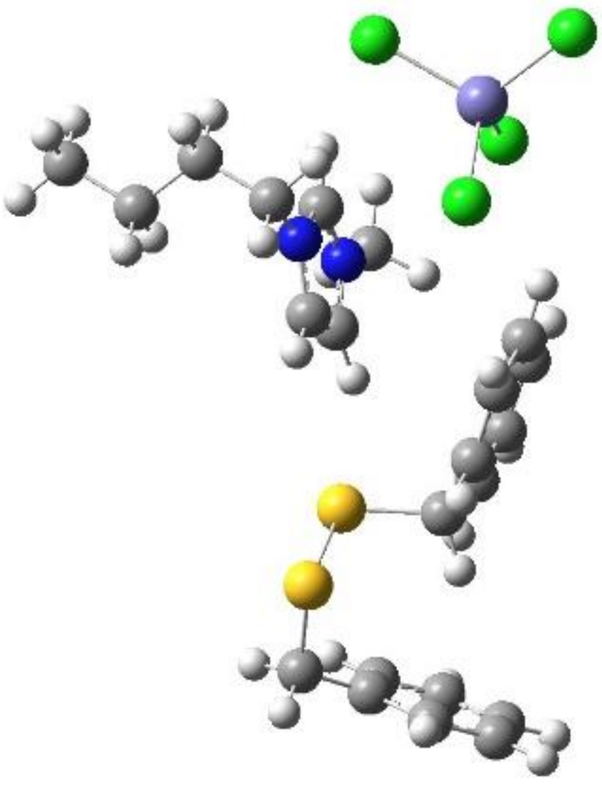

S3.2 [BMIM]BF_4-DBDS

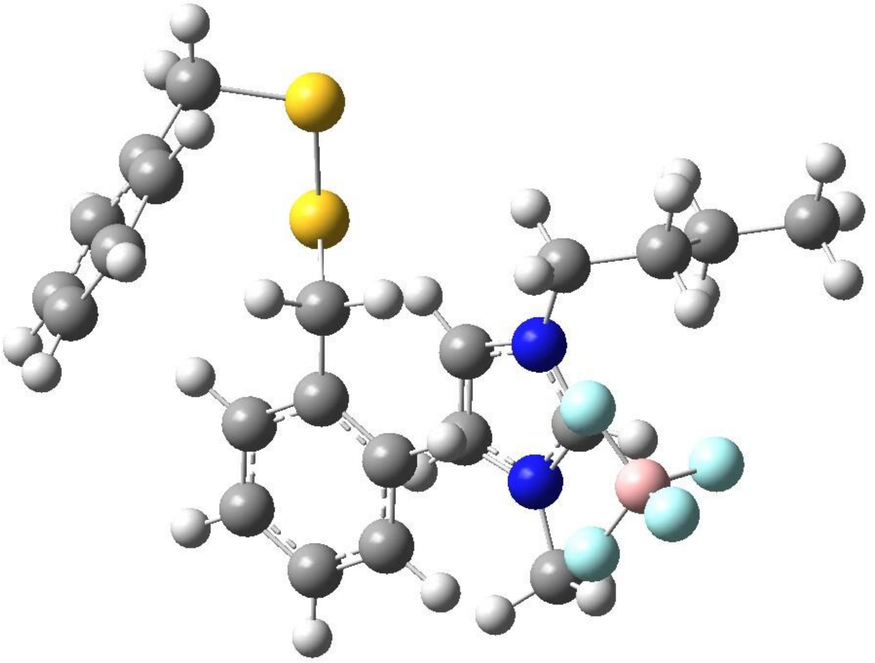

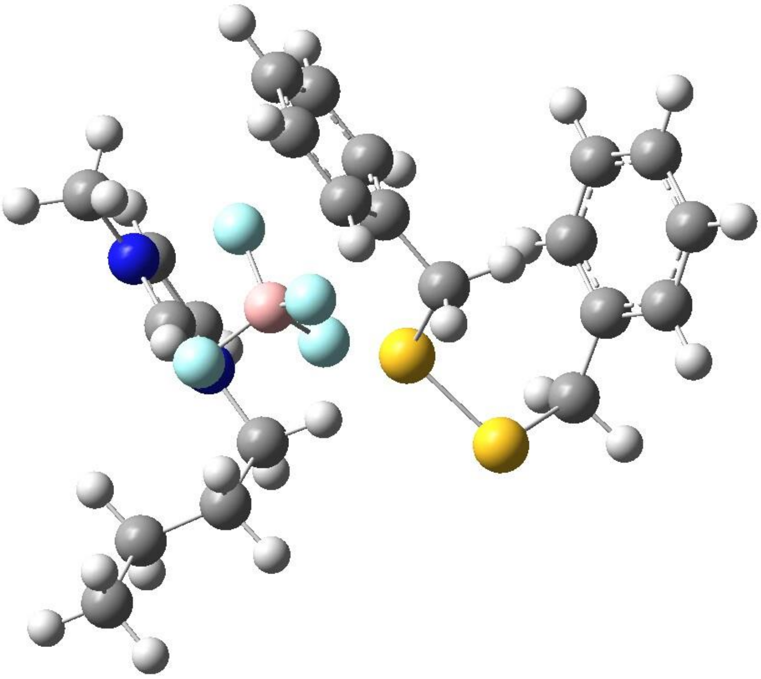

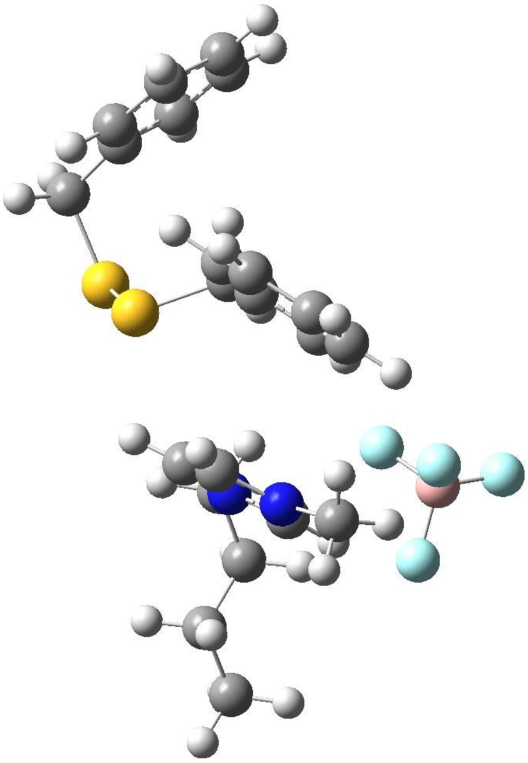

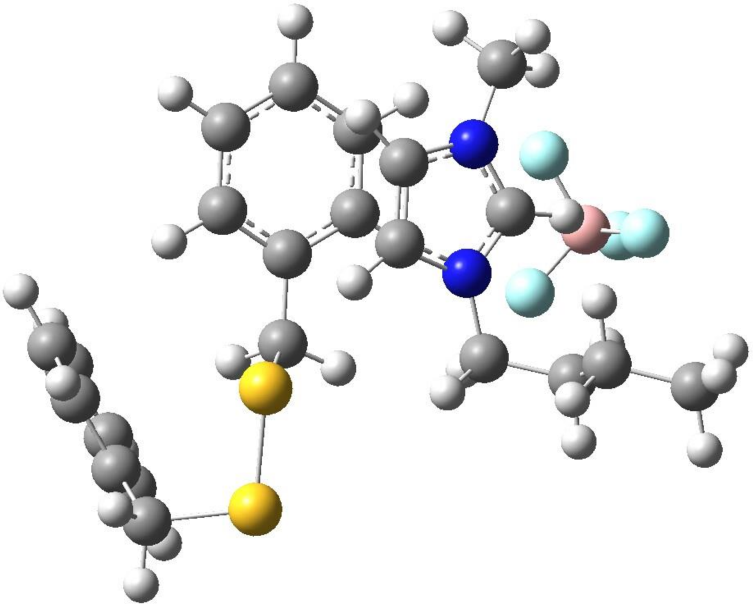

Supplement: Supplementary file 1 [file molecules-29-02395-s001.zip › molecules-2949929-supplementary.pdf]
